# Dietary Antioxidants and Lung Cancer Risk in Smokers and Non-Smokers

**DOI:** 10.3390/healthcare10122501

**Published:** 2022-12-10

**Authors:** Naser A. Alsharairi

**Affiliations:** Heart, Mind & Body Research Group, Griffith University, Gold Coast, QLD 4222, Australia; naser.alsharairi@gmail.com

**Keywords:** dietary antioxidants, vitamins, minerals, lung cancer, oxidative stress, lipid peroxidation, smokers, non-smokers

## Abstract

Smoking is considered a major risk factor in the development of lung diseases worldwide. Active smoking and secondhand (passive) smoke (SHS) are related to lung cancer (LC) risk. Oxidative stress (OS) and/or lipid peroxidation (LP) induced by cigarette smoke (CS) are found to be involved in the pathogenesis of LC. Meta-analyses and other case-control/prospective cohort studies are inconclusive and have yielded inconsistent results concerning the protective role of dietary vitamins C and E, retinol, and iron intake against LC risk in smokers and/or non-smokers. Furthermore, the role of vitamins and minerals as antioxidants with the potential in protecting LC cells against CS-induced OS in smokers and non-smokers has not been fully elucidated. Thus, this review aims to summarize the available evidence reporting the relationships between dietary antioxidant intake and LC risk in smokers and non-smokers that may be used to provide suggestions for future research.

## 1. Introduction

Smoking is the major cause of cancer mortality, responsible for 64.2% of global lung cancer (LC)-related deaths in 2019 [1]. There is clear evidence from longitudinal and/or case–control studies that support the link between current smoking and LC risk [2,3]. Secondhand smoke (SHS) as well as other environmental and genetic factors are potential risk factors for the development of LC in non-smokers [4,5]. Approximately 6% of global non-smoker deaths from LC in 2019 are caused by SHS exposure [1].

Tobacco smoke contains a mixture of toxic compounds including lung carcinogens, with tobacco-specific N-nitrosamines (i.e., NNN and NNK) considered to be potent carcinogens. These carcinogens can induce DNA adduct production in the lungs, thereby causing mutations in critical genes that could lead to the activation of key signaling pathways involved in the cellular processes of LC cells [6]. Although nicotine is not regarded as a carcinogen [7], it may contribute to the pathogenesis of LC, by binding nicotine-derived NNK to the homomeric α7 nicotinic acetylcholine receptor (α7nAChR), which results in significantly activated of α7nAChR-mediated cellular signaling pathways [8,9]. 

LC is comprised of two main histological types: small-cell lung cancer (SCLC) and non-small-cell lung cancer (NSCLC), with an incidence of 15% and 85% of LC cases respectively [10]. SCLC patients have worse health conditions than their NSCLC counterparts as evidenced by the presence of hematogenous metastases and comorbidities [11]. NSCLC is further classified into three phenotypes: squamous, adenocarcinoma, and large-cell carcinoma [10]. Squamous cell carcinoma (SCC) is more common in male smokers, while adenocarcinoma (ADC) predominates in female non-smokers [12,13,14,15]. LC in smokers is caused by several genetic/epigenetic changes. Smokers have higher mutation rates and frequency of genetic signatures of transversions than non-smokers [16]. The driver gene mutations in non-smoking-related LC differ from those in smokers. Kirsten rat sarcoma viral oncogene homolog (KRAS) mutations, epidermal growth factor receptor (EGFR) mutations, ROS Proto-Oncogene 1, Receptor Tyrosine Kinase (ROS1) and echinoderm microtubule–associate protein such as 4/anaplastic lymphoma kinase (EML4-ALK) fusion are the most gene mutations distinct in non-smokers, for which treatment strategies with medications to these mutations improved survival in ADC [16,17,18,19]. On the other hand, phosphatase and tensin homolog deleted on chromosome ten (PTEN) mutations, v-raf murine sarcoma viral oncogene homolog B1 (BRAF) mutations, discoidin Domain Receptor Tyrosine Kinase 2 (DDR2) mutations, protein 53 (p53) mutations, SRY-Box Transcription Factor 2 (SOX2) and fibroblast growth factor receptor (FGFR) have been identified as having the most driver genes in smoking-associated SCC [16,20].

Several therapies have been previously tested in randomised controlled trials (RCTs) and successfully approved for lung ADC patients [21]. Platinum-based chemotherapeutic agents (cisplatin and carboplatin) have been recently approved for the treatment of lung SCC and non-SCC in combination with anti-programmed death receptor 1 (PD-1) monoclonal antibodies (pembrolizumab, atezolizumab, bevacizumab and nivolumab) targeting T cell immune checkpoints [22,23]. Furthermore, the EGFR tyrosine kinase inhibitor (EGFR-TKI), osimertinib, dacomitinib, afatinib, gefitinib and erlotinib, have shown superior clinical survival benefits in lung SCC and/or ADC patients with EGFR mutation [24,25,26,27]. 

There is uncertainty about the benefits of dietary supplements, including vitamins and minerals, for LC prevention among smokers and non-smokers due to the inconsistency and contradictory results from RCTs [28]. There is also emerging concerns about the short and long-term safety of dietary supplements used by LC patients, particularly in smokers [29]. Therefore, there is a need to explore the effects of dietary antioxidants on LC risk in smokers and non-smokers. Dietary antioxidant vitamins and minerals are commonly derived from fruits, vegetables, cereals/grains, meat/meat products, oils and fat [30,31]. Smokers generally have lower diet quality than non-smokers, in terms of consuming dietary antioxidant vitamins and minerals [32,33]. Current and former smokers have lower circulating levels of antioxidant micronutrients (e.g., vitamins C and E, carotenoids) than non-smokers. The dietary differences observed between smokers and non-smokers may be due to results across studies that have been adjusted for different dietary patterns and other confounding variables in current/former smokers before and after quitting smoking [34]. Few meta-analyses have shown that dietary vitamin E and β-carotene intake reduce LC risk in smokers [35,36]. Evidence from observational studies and RCT suggests that dietary iron zinc, potassium and magnessium intake are associated with reduced LC risk [37,38,39]. Given that dietary antioxidant vitamins and minerals can have beneficial effects on reducing LC risk, targeting these nutrients might increase understanding of the potential mechanisms involved in such disease treatment, particularly in smokers. Thus, this review aims to summarize existing evidence regarding the role of dietary antioxidant vitamins (A, C, D, E, and β-carotene) and minerals (zinc, iron, copper and selenium) intake in LC prevention in smokers and non-smokers. It is hypothesized that dietary antioxidants play a significant role in reducing cigarette smoke (CS)-induced oxidative stress (OS) in LC smokers and non-smokers. This review does not address antioxidant vitamins and minerals bioavailability in foods, as this is extensively discussed in several other studies.

## 2. Methods

For this narrative review, the literature was searched via PubMed/MEDLINE and Google Scholar to retrieve English language studies up to October 2022 using the following keywords: LC, dietary antioxidants, vitamins, minerals, smoking, nicotine, current smokers, former smokers, non-smokers, OS, oxidative biomarkers and lipid peroxidation (LP). The search was primarily focused on the effects of dietary antioxidant vitamins and minerals intake on LC risk in smokers and non-smokers. The search also included relevant literature using selected keywords and was not restricted based on study design.

## 3. Associations between Dietary Antioxidants Intake and LC Risk in Smokers and Non-Smokers

### 3.1. Smokers

The findings from the meta-analyses and a few case-control/prospective cohort studies suggest that dietary carotenoids, vitamins E (tocopherols) and C (ascorbate) exert a protective effect against LC risk in current/former smokers. Such an effect may have also gender differences. Men current/heavy smokers have protection against LC risk from intake of β-cryptoxanthin (BCX), lycopene, and α/β-carotene, whereas women current smokers have protection from dietary vitamin C and retinol intake. A pooled analysis of population-based cohort studies investigating the associations between dietary carotenoid intake and LC risk, demonstrated that a high intake of BCX reduced LC risk by 30% among current smokers [35]. In a pooled analysis of prospective cohort studies, a significantly decreased LC risk by 26% was reported in current smokers who consumed vitamin E (α-tocopherol) [36]. A pooled analysis of case-control and cohort studies revealed no significant associations between a high intake of vitamin C and LC risk in current and former smokers [40]. In a case-control study that examined the associations between four dietary tocopherols obtained by a semi-quantitative food frequency questionnaire (FFQ) and LC risk found that a high intake of α-tocopherol was associated with reduced risk of LC in current and former smokers compared to healthy controls [41]. A longitudinal population-based study examined the association between dietary antioxidant intake measured by a 24-h recall and LC risk and showed that a high intake of vitamin E, vitamin C and carotenoids were associated with reduced LC risk in current smokers with ≤33 packs/year [42]. A longitudinal study that investigated the association between dietary vitamin intake assessed using an FFQ and LC risk found that the association of retinol intake was positively significant in male current smokers, and negatively significant in female current smokers. The intake of α-carotene was associated with reduced LC risk in male light smokers [43]. A high intake of BCX, lycopene and α/β-carotene was associated with a reduced risk of LC in male heavy-intensity smokers, while a high intake of vitamin C was associated with a reduced risk in female heavy-intensity smokers [44].

Only a few case-control and prospective cohort studies have suggested that dietary zinc and copper intake are associated with reduced LC risk in current smokers, but the results of iron intake have been inconsistent. A study aimed to investigate the association between dietary mineral intake assessed by semi-quantitative FFQ and LC risk, found that among current smokers, zinc intake was associated with a lower risk of LC, whereas iron intake was associated with a higher risk of LC [45]. A case-control study found that zinc and copper intake among current smokers compared with healthy controls was associated with a reduced risk of LC. However, selenium intake was not associated with LC risk in current/former smokers [46]. A prospective cohort study revealed an inverse association between total iron intake (mg/1000 kcal), but not haem and non-haem iron intake, assessed by semi-quantitative FFQs, and LC risk among former smokers only [47].

### 3.2. Non-Smokers

The findings from case controls and prospective cohort studies suggest vitamins E and D and β-carotene may reduce the risk of LC in non-smokers, while retinol had no effect. A population-based cohort study showed that consumption of β-carotene, but not retinol, was associated with a decreased risk of LC [48]. Another longitudinal study found that a high dietary α-tocopherol intake measured by an FFQ was associated with a reduced risk of LC in non-smokers exposed to passive smoke at home and work [49]. A pooled analysis of case-controls and prospective cohort studies reported a 24% decrease in LC risk after vitamin D consumption in non-smokers [50].

The effects of dietary antioxidant vitamins and minerals on LC risk in smokers and non-smokers are summarized in Table 1. 

## 4. Cigarette Smoke-Induced Oxidative Stress in Lung Cancer 

OS is considered the key mechanism responsible for the development of smoking-related LC. Levels of OS and LP were found to be higher, and antioxidant enzymes and vitamins/minerals were lower in current and former smokers than non-smokers. A recent review suggests that there are lower plasma levels of β-carotene, BCX, lycopene, vitamin E, vitamin C and selenium in current smokers than non-smokers [51]. In one cross-sectional study, female current, but not former smokers have higher levels of urinary 8-hydroxy-20-deoxyguanosine (8OHdG), a biomarker for CS-induced oxidative damage, than their male counterparts; although they had elevated serum levels of β-carotene and BCX. This is possibly due to low levels of erythrocyte superoxide dismutase (SOD), an antioxidant enzyme that degrades superoxide free radicals, thereby increasing oxidative DNA damage and driving gene mutation in LC cells [52]. SOD activity was found to be lower in male heavy smokers compared to male non-smokers [53]. In a case-control study that examined the effects of CS on salivary antioxidant activity found that SOD activity was lower in male current smokers than non-smokers [54]. Results from a case-control study showed that current smokers have higher nitrite/nitrate, carbonyl and iron levels, erythrocytes nitric oxide synthase (NOS) protein expression, and lower plasma vitamin C and glutathione (GSH) enzyme, compared to non-smokers [55]. In a case-control study of antioxidant activity in erythrocytes of NSCLC cases compared with healthy controls, SOD activity was found to be lower, while glutathione peroxidase (GPx) was found to be higher in current and non-smokers [56]. Evidence from an RCT revealed an increase in the urinary 8OHdG levels of men and women current smokers compared to their non-smoker counterparts [57]. Experimental studies showed that high levels of 8-OHdG and low levels of antioxidant capacity (AOC) were associated with non-smoking patients with NSCLC compared to smokers [58,59]. High urinary 8-OHdG levels have been reported in non-smokers [60]. An experimental study suggested that an oxidant and antioxidant imbalance exists in smokers as compared with non-smokers. Results showed that the serum malondialdehyde (MDA) level, a lipid peroxidation product assessed as a biomarker of OS, is increased, whereas the serum paraoxonase 1 (PON1) level, an antiatherogenic enzyme that hydrolyzes oxidized lipids and paraoxon in Clara cells of the lung is decreased in current smokers compared with those of non-smokers [61]. 

**Figure 1:**
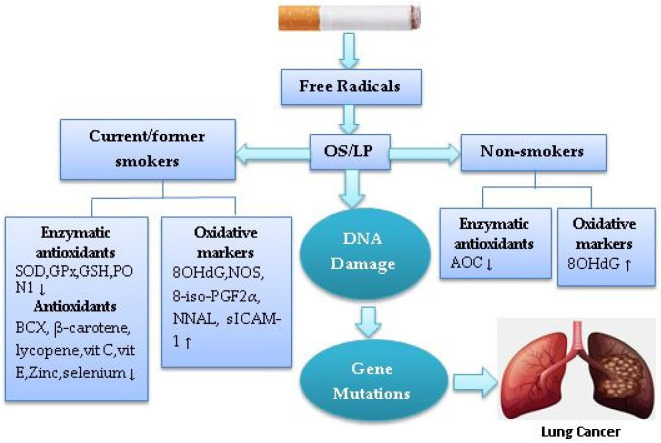
The schematic diagram of cigarette smoke-induced oxidative stress in lung cancer. (↓) decrease, (↑) increase.

A meta-analysis of 18 experimental studies showed that the levels of urinary isoprostane-8-iso prostaglandin F_2α_ (8-iso-PGF_2α_), a biomarker of OS/LP, are higher in current smokers than non-smokers [62]. A case-control study showed evidence of associations between urinary 8-epiPGF 2α and LC risk in both current and former smokers, but current smokers displayed higher levels of 8-epiPGF 2α than former and non-smokers [63]. Current smokers have significantly higher fasting serum levels of nitric oxide (NO) than non-smokers [64]. A cross-sectional study of CS-induced OS biomarkers showed that current smokers have higher urinary 8-iso-PGF2α Type III, nicotine equivalents and total 4-(methylnitrosamino)-1-(3-pyridyl)-1-butanol (NNAL) levels than former and non-smokers [65]. Another cross-sectional study of OS/inflammatory biomarker levels by tobacco user groups showed that smokeless tobacco (ST) users among former and non-smokers had high serum levels of F2-isoprostane, soluble intercellular adhesion molecule-1 (sICAM-1) and interleukin (IL)-6 [66]. There is cross-sectional evidence that the lungs of electronic cigarette (e-cig) users had higher levels of inflammatory biomarkers (IL-6, IL-1β) than non-smokers, but lower levels than those of current smokers [67]. LC current and former smokers demonstrated higher 8OHdG levels and lower plasma vitamin E levels than non-smokers [68]. It can be suggested that smoking increases OS in both smokers and non-smokers, but current smokers may be more susceptible to CS-induced OS than former and non-smokers. Figure 1 shows that CS induces OS/LP in smokers and non-smokers. 

## 5. Potential Role of Dietary Antioxidants against Cigarette Smoke-Induced Oxidative Stress in Lung Cancer 

### 5.1. Antioxidant Vitamins

#### 5.1.1. Retinol

Retinol, retinal or retinoid acid (RA), which is derived from protein/animal sources (e.g., chicken, egg), may explain the association with LC risk in smokers [43]. RA was reported to promote inflammation-induced oxidative DNA damage in NSCLC cell line A549 in smokers by suppressing hydrogen peroxide (H2O2)-utilizing myeloperoxidase (MPO) enzyme produced by activated neutrophils, thereby increasing reactive oxygen species (ROS) and hydroxyl radical production [69]. Nicotine has been shown to suppress the growth inhibitory effects of trans-RA in NSCLC cell line H460 by inhibiting its ability to enhance RA receptor beta (RARβ) expression due to the increase an orphan RA receptor TR3 expression, suggesting that RA may be ineffective in reducing LC risk in smokers [70]. Bexarotene (Targretin, LGD1069), a synthetic retinoid, has demonstrated significant inhibition of the metastasis and angiogenesis of NSCLC cells by suppressing vascular endothelial growth factor (VEGF) and metalloproteinase (MMP) expression through downregulation of the α7nAChR-mediated c-JUN NH2-terminal kinase/extracellular signal-regulated kinase (JNK/ERK) signaling pathway [71]. Treatment with RA in combination with ERK inhibitors may suppress the migration of NSCLC cells in vitro [71]. This review showed that dietary retinol intake was associated with increased LC risk in male current smokers. Such an association may be due to the interaction of retinol and the CS in LC cells, resulting in a reduction of RAR-β expression and activation of proliferative markers. However, the results demonstrated a reduction in LC risk among female current smokers with dietary retinol intake. The reasons for the contrast of such an association require further clarification as to possible mechanisms.

#### 5.1.2. β-Cryptoxanthin and Lycopene

BCX and lycopene, which are commonly derived from fruits, may play a vital role as LC preventive agents in smokers. BCX exerts anti-proliferative and apoptotic/autophagic effects on nicotine-induced A549 cells in vitro by induction of G1/G0 phase arrest via inhibiting cyclinD1/E and upregulating P21 expression [72]. BCX also exerts a protective effect against NNK-induced lung tumours in vivo by inhibiting the protein and mRNA levels of the α7nAChR via downregulating the α7nAChR/phosphatidylinositol-3 kinase (PI3K) signaling pathway [73]. In vivo, BCX supplementation has been shown to inhibit CS exposure-induced lung inflammation, as evident by reducing the levels of 8OHdG, tumour necrosis factor-α (TNFα), nuclear transcription factor-kappaB (NF-κB) and activator protein-1 (AP)-1 in lung macrophages [74]. Another in vivo study showed that BCX exerts protective effects against LC and emphysema by reducing nicotine/NNK-induced Sirtuin 1 (SIRT1) mRNA, reducing RARβ and p53 mRNA levels and increasing serine-threonine kinase (AKT) phosphorylation levels [75]. Treatment of A549 cells with lycopene resulted in inhibition of CS-induced OS in vitro by enhancing connexin-43 (Cx43) protein, 8-oxoguanine DNA glycosylase (OGG1) and Nei-like DNA glycosylases (NEIL1, NEIL2, NEIL3) levels, suggesting that the carotenoid lycopene may have therapeutic potential in smoke-induced LC by modulating the expression of multiple DNA repair genes [76]. Treatment with lycopene also triggers a protective effect against LC in vivo by inhibiting NNK-induced α7nAChR expression [77]. Lycopene exerts antioxidant effects in vivo by improving pulmonary emphysema by reducing CS-derived MPO/NO-induced LP/DNA damage and increasing erythrocyte SOD and GPx activity. It also exerts anti-inflammatory effects by reducing TNF-α, interferons (IFN-γ) and IL-10 cytokine production [78]. High dietary lycopene intake was shown to suppress CS/NNK-induced emphysema in vivo by reducing mRNA expression of ATP-binding cassette transporter G1 (ABCG1), liver X receptor-α (LXRα) and peroxisome proliferator-activated receptor α (PPARα) involved in reverse cholesterol transport, which is the main driver of persistent and excessive inflammation after CS exposure [79]. This suggests that lycopene and BCX may play a significant role in reducing LC risk in smokers.

#### 5.1.3. β-Carotene, Vitamins C and E 

Several studies of vitamin supplements (e.g., vitamins C and E, β-carotene) provide contradictory results for protection against LC in smokers [28]. Limited evidence from RCTs showed that supplementation with β-carotene increases LC risk and mortality among current smokers [28]. β-carotene is considered an antioxidant-beneficial agent in human and animal lung tissues due to its ability to reduce CS-induced LP. However, their benefits may shift from being an antioxidant to a prooxidant causing oxidative DNA damage in lung tissue when exposed to tobacco smoke condensate via a range of mechanisms including downregulating RA signaling, elevating markers of OS, increasing molecular expressions involved in cellular proliferation/apoptosis, and the production of carotenoid oxidation products [80]. An in vitro experimental study showed that β-carotene does not promote LP levels in liposomal membrane tissue caused by smoking or reduce other antioxidant vitamins such as α-tocopherol and ascorbate. β-carotene, the so-called non-polar carotenoid, was found to be sensitive to CS-induced oxidation and its levels in methanol were depleted in response to CS exposure [81]. β-carotene is sensitive to CS-dependent oxidation in such a way that it can react with a mixture of nitrogen oxides (NO2), peroxynitrite and volatile peroxyl radicals to generate a β-carotene radical via the synthesis of the cation radical [82,83]. It can be suggested that, although β-carotene is oxidized by smoke, it is unlikely to increase LC risk in smokers. The treatment of pneumocyte cells with β-carotene and its short-chained volatile aldehydes in the presence of oxidative stress resulted in a significant decrease in DNA damage even at a concentration of 50μM [84], suggesting that β-carotene acts as an antioxidant and may have a protective effect against LC in smokers. 

β-carotene, α-tocopherol and ascorbic acid have been shown to attenuate CS-induced NO2 and aldehydes, which cause biomolecular damage enhancing LP through free radical mechanisms [85]. β-carotene and vitamin E levels in heavy smokers were associated with lung function decline in longitudinal 8 years of follow-up, suggesting that such antioxidants may protect lung tissue by reducing OS induced by CS [86]. Low plasma α-tocopherol and ascorbic acid levels have been observed in smokers more than non-smokers [87]. In an in vitro experimental study, vitamin C was been shown to reduce aqueous extract of CS-induced A549 cell proliferation by deactivating p-benzoquinone, thereby downregulating EGFR and its downstream molecules, suggesting that vitamin C may have potential as an antioxidant for reducing LC risk in smokers [88].

Antioxidant supplementation with vitamins C, E and β-carotene resulted in significantly decreased urinary 8OHdG and carbonyl levels in current smokers [89]. Vitamin C and E supplementation were found to be effective in reducing 8-epiPGF_2α_ in the plasma of current smokers [90]. A randomized placebo-controlled trial resulted in decreased urine 8-iso-PGF_2α_ by 21% in male current smokers after receiving 400IU/d of vitamin E (rac-α-tocopheryl acetate) over 36 months compared to those in the placebo group (no vitamin E supplementation) [91]. An RCT which examined the effect of antioxidant vitamins consumption on LP in male smokers showed a significant reduction in plasma MDA levels over one month of vitamin supplementation (500 mg vitamin C, 9 µg β-carotene, 200 IU vitamin E) compared to non-smokers [92]. Vitamin E supplementation has been shown to improve lung function in current smokers by reducing plasma MDA levels and increasing SOD and GPx activity [93]. A case-control study showed that supplementation with antioxidant vitamins (60 mg vitamin C, 3000 µg β-carotene, 30 IU vitamin E) over 60 days decreased 8-oxodGuo by 62% in groups exposed to tobacco smoke compared with controls [94]. 

A few cohort studies have demonstrated a reduction in LC risk among male smokers receiving α-tocopherol supplementation [95,96]. CS oxidizes α- and γ-tocopherol, leading to reduced plasma levels in smokers compared with non-smokers [97,98], and supplementation with ascorbic acid could have a significant effect in decreasing α-and γ-tocopheroxyl free radicals to nonoxidized forms, thereby increasing tocopherol plasma levels [98]. A placebo-controlled parallel intervention study showed a reduction in oxidative DNA damage in mononuclear blood cells in male smokers exposed to the vitamin C (500 mg/d) and vitamin E (182 mg/d) intervention over a four week period compared to those in the placebo group (no vitamin C or E supplementation) [99]. In another double-blind, placebo-controlled investigation, male smokers received either the daily dietary supplement (31 mg all-rac-α-tocopheryl acetate 272 mg vitamin C) or placebo (non-digestible carbohydrate and <2% stearic acid) for three months. Results showed that plasma α-tocopherol levels were increased in both groups, while plasma vitamin C levels were only significantly repleted in the intervention group [100]. 

One RCT study suggests that high-dose multiple antioxidant vitamins (1050 mg/day vitamin E, 6100 mg/day ascorbic acid and 60 mg/day β-carotene) used in combination with chemotherapy (carboplatin and paclitaxel) failed to protect NSCLC cells from free radical damage [101]. Treatment of H460 cells with the chemotherapeutic drug paclitaxel in the presence of α-tocopheryl succinate, a powder form of α-tocopherol, showed apoptotic effects through increased poly ADP ribose polymerase (PARP) and caspase 8 expression [102].

Taken together, β-carotene and vitamins C and E appear to have protective effects against CS-induced OS in LC smokers. Supplementation with these antioxidants resulted in reduced OS biomarkers. Vitamin E in combination with chemotherapy may have useful apoptotic effects against nicotine-induced NSCLC.

This review demonstrated a reduction in LC risk among male current smokers with a high intake of dietary carotenoids (β-carotene, BCX and lycopene), and female current smokers with a high intake of vitamin C. High dietary intake of β-carotene and vitamin E was associated with reduced LC risk in non-smokers, while high dietary intake of vitamin E only was associated with reduced risk of LC in both current and former smokers. The underlying reasons for such effects are unclear. Given that smokers and non-smokers are susceptible to CS-induced OS, but current and former smokers to higher levels than non-smokers, dietary carotenoids, vitamins C and E may play a significant role in protecting lung cells against CS-derived free radicals/oxidant-induced oxidative damage in LC. Further studies are needed to explore gender differences in dietary antioxidant vitamins intake and LC risk, and how these relationships may relate to smoking status. 

#### 5.1.4. Vitamin D 

Vitamin D as a potent antioxidant has been implicated in reducing NSCLC risk via α7nAChR-mediated cellular signaling pathway inhibition, but there is no evidence to date on its association with reducing CS-induced OS/LP in LC smokers and non-smokers. A meta-analysis of 24 case-control and cohort studies showed that smoking was associated with lower blood 25(OH)D levels in smokers and non-smokers [103]. In a longitudinal cohort study with follow-up over 20 years, vitamin D deficiency was found to be associated with lung function decline in current smokers [104]. In vivo, vitamin D has been shown to protect lung cells from CS-induced oxidative damage [105]. A randomized, double-blind trial demonstrated significantly improved survival of lung ADC patients with lower 25(OH)D levels receiving vitamin D supplements (1200 IU/day) over a one-year period compared with those in placebo groups (not taking a supplement) [106]. In vitro, vitamin D suppresses proliferation/invasion and induces apoptosis of A549/NCI-H1975 cells, and enhances the sensitivity of these cells to the chemotherapeutic drug cisplatin. Such an effect is mediated by downregulating the PI3K/AKT/mammalian target of the rapamycin (mTOR) signaling pathway [107]. Treatment with platinum-based doublet first-line chemotherapy resulted in a significant reduction of plasma vitamin D (25(OH)D) levels in advanced NSCLC patients [108]. This suggests that vitamin D could be beneficial alone or in combination with chemotherapy to reduce LC risk in smokers. In this review, the preventive effect of vitamin D was observed in LC non-smokers exposed to passive smoke. The reason underlying this is that non-smokers might benefit from eating a diet rich in vitamin D, which could mitigate CS-induced OS, given that non-smokers are susceptible to CS-induced OS but to a lower extent than smokers.

### 5.2. Antioxidant Minerals

#### 5.2.1. Iron

The high intake of iron is not advocated as it results in significant increases in CS-induced OS in LC smokers. A recent meta-analysis of 20 prospective and case-control studies which examined the associations between different iron biomarkers/iron intake and LC risk, showed that transferrin saturation and serum ferritin high levels were associated with LC risk, whereas iron intake (heme iron, non-heme iron or total iron) had no effect [109]. High dietary iron intake has been shown to increase urinary MDA and cotinine levels in men and women exposed to CS [110]. It has been found that the 8-isoprostane levels were increased among smokers with a high iron intake [111]. High levels of iron in LC cells not only induce oxidative DNA damage but also ferroptosis, a novel iron-dependent type of regulated necrosis, caused by the accumulation of ROS through LP [112]. Cigarette smoke extract exposure upregulates heme oxygenase-1 (HO-1) expression in human lung cells [113,114], an enzyme responsible for catalyzing the degradation of exogenous heme to produce biliverdin, carbon monoxide and ferrous ions, in parallel with a reduction in GSH, resulting in induced ferroptosis and increased deca-pentaplegic homolog (Smad)-dependent pathways (e.g., c-Fos and c-Jun) expression [114]. In vivo, NNK has been shown to induce HO-1 expression in LC cells via the upregulation of NF-_κ_B and ERK1/2 signaling pathways [115]. Results obtained from this review demonstrated that a reduction in LC risk was associated with total iron, but not with haem and non-haem intake in former smokers. Such an association is inconsistent with the fact that iron intake increases LC risk in smokers. The mechanism behind such an effect is largely unknown. It is hypothesized that lymphoid-specific helicase (LSH), a DNA methylation modifier stabilizing the transcripts that enhances tumourigenesis in NSCLC [116], reduces CS-induced ferroptosis by interacting with stearoyl-coa desaturase-1 (SCD-1), which is an iron-containing microsomal enzyme which exists in the endoplasmic reticulum responsible for the degradation of saturated fatty acid into monounsaturated fatty acid [117]. The reduction of SCD-1 expression inhibits proliferation and induces apoptosis in NSCLC cells through reducing α7nAChR-mediated Akt phosphorylation [118]. Thus, LSH and its ferroptosis-related gene SCD-1 exert anti-tumour effects, which may reduce CS-induced ferroptosis by reducing intracellular haem and non-haem levels in NSCLC cells.

#### 5.2.2. Copper, Zinc and Selenium

There is controversy over the effects of copper on LC risk in smokers, whereas zinc and selenium could be beneficial in reducing the risk. This review demonstrated that a high intake of dietary zinc and copper was associated with a reduction in LC risk in current smokers. On the other hand, selenium intake had no effect on LC risk in smokers. A few meta-analyses of case-control studies have shown higher serum copper levels but lower serum zinc levels in LC patients than among healthy controls [119,120]. In vitro, treatment of human erythrocytes with hydroquinone, an ingredient of cigarette smoking, alone or in the presence of copper, increases H2O2 production and reduces GSH levels. This may be due to the binding of copper to the thiol group of reduced GSH or to the 1, 4 benzoquinone-GSH adduct production, suggesting that copper may increase erythrocyte membrane OS in smokers [121]. In a case-control study, an association between the plasma levels of copper and LP, assessed as fluorescent damage products of LP was observed in greater proportions among smokers as compared to non-smokers [122]. An in vitro study showed that treatment of A549 cells with the aqueous almond skin extract-capped copper nanorods reduced antioxidant enzyme GPx activity and mitochondrial membrane functions causing induction of cellular OS by increasing ROS production in high quantities [123].

**Figure 2:**
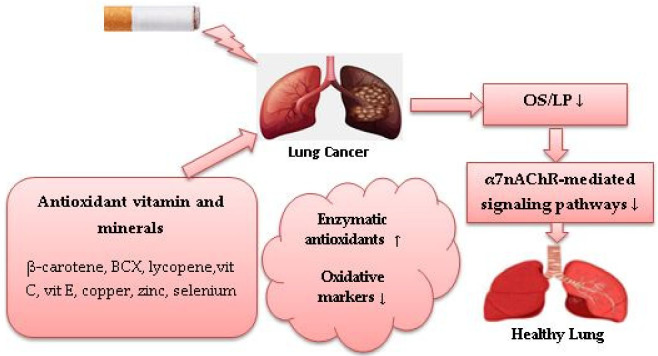
Role of dietary antioxidants against cigarette smoke-induced oxidative stress in lung cancer. (↓) decrease, (↑) increase.

In a case-control study that examined associations between serum trace elements status and redox status parameters in LC patients compared with healthy controls, zinc supplementation was positively associated with total antioxidant status and negatively associated with total oxidant status. Results of that study also demonstrated that copper and Cu:Zn ratios were positively associated with SOD activity and negatively associated with catalase activity [124]. An experimental study demonstrated apoptosis induction on A549 cells in vitro when treated with zinc in different concentrations in combination with docetaxel (Taxotere), a semisynthetic antineoplastic agent used for NSCLC treatment, through upregulating expression of p53. Such an effect is accompanied by an increase in GSH and GPx activity [125]. An experimental study that investigated associations between plasma mineral levels and erythrocyte antioxidative enzyme activity in smokers, demonstrated a positive association between consumption of selenium and GPx, copper and copper-zinc superoxide dismutase (CuZnSOD), and a negative association between selenium and plasma thiocyanate levels used as an indicator of smoking status [126]. In an in vivo experimental study, CuZnSOD has been shown to reduce oxidative damage and LP in the lungs of mice in response to CS exposure. Results also showed that treatment with SOD causes a significant reduction in the number of A549 cells/macrophages and inhibition of neutrophil chemotaxis following CS exposure [127]. Evidence showed that smokers have higher serum thiobarbituric acid reactive substance levels, which are used as an indicator of CS-induced OS, but lower serum selenium GSH and GPx levels than non-smokers [128]. In vivo, selenium pretreatment of rats exposed to CS showed a significant reduction in the plasma LP, total cholesterol levels and triacylglycerol [129]. A case-control study showed that supplementation with 40 µg selenium, 40 mg zinc and 2 mg copper over 60 days reduced 8-oxodGuo levels in smokers compared with non-smokers [94]. In one experimental in vitro study, treatment with copper alone or in combination with the chemotherapeutic drug disulfiram induced G2/M cell cycle arrest in A549 and NCI-H2009-cells and reduced the mRNA expression of NSCLC-related genes [130]. These findings suggest that zinc and selenium could be helpful in protecting against CS-induced OS in LC smokers. Copper in combination with chemotherapeutics or supplemented with zinc and selenium could be beneficial for reducing CS-induced OS in LC current smokers. Increased CuSOD activity may have a potential antioxidant effect against LC risk in smokers by reducing CS-induced OS/LP. Figure 2 shows the role of dietary antioxidant vitamins and minerals against CS-induced OS in LC.

## 6. Conclusions 

The diet quality of smokers compared to non-smokers, in terms of consuming antioxidant vitamins and minerals, is relatively poor. OS has been shown to be a potential contributing factor in the pathogenesis of CS-induced LC risk. The serum/plasma levels of β-carotene, BCX, lycopene, vitamin E, vitamin C, vitamin D, zinc and selenium have been shown to be lower in LC smokers when compared to non-smokers. This indicates that low levels of these antioxidants may be associated with increased CS-OS biomarkers in smokers, which in turn are risk factors for LC. 

Interventional studies showed that supplementation with β-carotene, vitamin E, vitamin C, copper, zinc and selenium could reduce serum/plasma levels of CS-OS biomarkers in LC smokers. However, iron has an oxidant effect, which makes it difficult to recommend as a supplement for LC treatment in smokers in view of its association with CS-induced OS. 

Findings from this review support the view that dietary antioxidant vitamins (C, D, E and carotenoids) and minerals (zinc and copper) have a protective role against LC risk, and support the hypothesis that these antioxidants could be effective in protecting LC cells from CS-induced OS/LP in LC smokers and non-smokers. However, the mechanisms by which these antioxidants might be exerting their protective effects against LC risk have not been fully elucidated. The effect of dietary antioxidant vitamins and minerals on LC is still inconclusive, as dietary vitamins C and E, retinol and iron differentially affect LC risk in smokers. Retinol may be effective in reducing LC risk in current smokers if it is used in combination with ERK inhibitors. Copper supplementation in combination with chemotherapeutics or supplemented with zinc and selenium could have a protective effect against LC risk in smokers. The increased activity of CuZnSOD may be effective as an LC treatment in smokers, but further studies into possible mechanisms behind such effects are needed.

Studies are still needed to investigate the protective effects of antioxidant vitamins and minerals intake on LC smokers and non-smokers. Future directions for exploring possible mechanisms underlying such effects are warranted. Further RCTs are needed to examine whether a high dietary intake of antioxidant vitamins and/or minerals in conjunction with chemotherapy may reduce CS-induced OS/LP in LC smokers and non-smokers. 

Further studies are also needed to determine whether retinol intake in female current smokers, α-carotene in male current smokers and iron intake in former smokers could protect against CS-induced OS and the subsequent development of LC. The role of gender in dietary antioxidant vitamins intake and LC risk, and how these associations may relate to smoking status, needs to be addressed in future studies.

## Figures and Tables

**Table 1:** Dietary antioxidants and the risk of LC in smokers and non-smokers.

Reference	Country	Research Design	Sample	Dietary Antioxidants	Findings
Männistö et al. [35]	Europe and North America	Meta-analysis of seven population-based cohort studies (follow-up duration of 7–16 years across studies)	Total subjects = 3155 LC patients Current smokers = 1915 Former smokers = 981 Non-smokers = 259	Carotenoids	High intake of BCX (≥160 µg/day) was associated with reduced LC risk in current smokers (RR = 0.70, 95% CI = 0.60 to 0.81)
Zhu et al. [36]	US, Netherlands, China, Finland and Denmark	Meta-analysis of nine population-based cohort studies (follow-up duration of 4–20 years across studies)	Total subjects = 2768 LC patients Current smokers = 1930 Former smokers = 528 Non-smokers = 310	Vitamin E (α-tocopherol)	Vitamin E intake (2 mg/day) was associated with reduced LC risk in current smokers (RR = 0.74, 95% CI = 0.61 to 0.89)
Luo et al. [40]	US, China, Netherlands, Uruguay and Canada	Meta-analysis of seven case-control and 14 population-based cohort studies	Total subjects = 2008 LC patients Current smokers = 1044 Former smokers = 702 Non-smokers = 262	Vitamin C	High intake of vitamin C (100 mg/day) was not associated with LC risk in current/former smokers
Mahabir et al. [41]	US	Case-control study	Total subjects = 2502 LC cases = 1088 (current smokers = 438; former smokers = 417; non-smokers = 238) Healthy matched controls = 1414 (current smokers = 512; former smoker = 599; non-smokers = 303) Age = ≥60 years	Dietary tocopherols (α-, β-, γ-, and δ)	High intake of α-tocopherol (≥5.51 mg/day) was associated with reduced LC risk in current smokers (RR = 0.33, 95% CI = 0.18 to 0.62) and former smokers (RR = 0.46, 95% CI = 0.26 to 0.81)
Yong et al. [42]	US	Population-based cohort study (follow-up duration of 19 years)	Total subjects = 10,068 Current smokers = 3090 Former smokers = 1691 Non-smokers = 4261 Unknown smoking status = 1026 Age = 25–74 years	Vitamins A, C and E, carotenoids	High intake of carotenoids (>2289.87 IU/day) (RR = 0.49, 95% CI = 0.29 to 0.84), vitamin E (>6.71 mg/day) (RR = 0.36, 95% CI = 0.16 to 0.83) and vitamin C (>113.05 mg/day) (RR = 0.55, 95% CI = 0.32 to 0.95) were associated with reduced LC risk in current smokers
Narita et al. [43]	Japan	Population-based cohort study (average 15.5 years follow-up)	Total subjects = 1896 LC patients Current smokers (male = 641, female = 28) Former smokers (male = 109, female = 0) Non-smokers (male = 109, female = 289) Light smokers (male = 37, female = 0) Heavy smokers (male = 713, female = 0) Age = 40–69 years	Retinol, vitamin C, vitamin E and carotenoids	Retinol intake (10 mcg/day) was associated with increased LC risk in male current smokers (HR = 1.22, 95% CI = 0.99 to 1.50), and decreased LC risk in female current smokers(HR = 0.51, 95% CI = 0.18 to 1.40) α-carotene intake (>2064 µg/day) was associated with reduced LC risk in male light smokers (OR = 0.29, 95% CI = 0.09 to 0.93) No other associations with LC were observed
Shareck et al. [44]	Canada	Case-control study	Total subjects = 2554 LC cases = 1105 (Male current smokers = 465; former smokers = 69; non-smokers = 156, Female current smoker = 304; former smokers = 36, non-smokers = 75) Healthy matched controls = 1449 (Male current smokers = 249; former smoker = 81; non-smokers = 540, Female current smokers = 127; former smokers = 48; non-smokers = 404) Age = 35–75 years	Vitamin C and carotenoids	High intake of **BCX** (>178 µg/day) (OR = 0.58, 95% CI = 0.34 to 0.89), β-carotene (>6760 µg/day) (OR = 0.49, 95% CI = 0.28 to 0.83), α-carotene (>2064 µg/day) (OR = 0.53, 95% CI = 0.31 to 0.89) and lycopene (>19,281 µg/day) (OR = 0.48, 95% CI = 0.29 to 0.79) were associated with reduced LC risk in male heavy-intensity smokers High intake of vitamin C (≥79 mg/day) (OR = 0.45, 95% CI = 0.21 to 0.95) was associated with reduced LC risk in female heavy-intensity smokers
Zhou et al. [45]	US	Case-control study	Total subjects = 2048 LC cases = 923 (current smokers = 378; former smokers = 489; non-smokers = 56) Healthy matched controls = 1125 (current smokers = 213; former smoker = 518; non-smokers = 394) Age = ≥18 years	Iron and zinc	Iron intake (≥16.24 mg/d) was associated with increased LC risk in current smokers (OR = 4.03, 95% CI = 1.89 to 8.75) Zinc intake (≥12.88 mg/d) was associated with reduced LC risk in current smokers (OR = 0.41, 95% CI = 0.19 to 0.88)
Mahabir et al. [46]	US	Case-control study	Total subjects = 3352 LC cases = 1676 (current smokers = 747; former smokers = 693; non-smokers = 256) Healthy matched controls = 1676 (current smokers = 584; former smoker = 779; non-smokers = 313) Age = ≥60 years	Zinc, copper and selenium	Zinc intake (>12.31 mg/d) was associated with reduced LC risk in current smokers (OR = 0.36, 95% CI = 0.22 to 0.57) Copper intake (>1.56 mg/d) was associated with reduced LC risk in current smokers (OR = 0.38, 95% CI = 0.24 to 0.60) Selenium intake was not associated with LC risk
Ward et al. [47]	Denmark, Greece, Italy, France, Germany, Netherlands, Norway, Spain, Sweden and the UK	Multi-centre prospective cohort study (follow-up duration of 8 years)	Total subjects = 83,348 Current smokers = 21,754 Former smokers = 20,171 Non-smokers = 41,423 Age = ≥49 years	Iron	Total iron intake (mg/1000 kcal), but not haem and non-haem iron intake was associated with reduced LC risk in former smokers only (HR = 0.90, 95% CI = 0.83 to 0.97)
Mayne et al. [48]	US	Population-based cohort study (follow-up duration of 3 years)	Total subjects = 413 non-smokers Age = ≥60 years	β-carotene and retinol	β-carotene intake was associated with reduced LC risk (OR = 0.70, 95% CI = 0.50 to 0.99) Retinol intake was not associated with LC risk
Wu et al. [49]	China	Population-based cohort study (follow-up duration of 12 years)	Total subjects = 72,829 female non-smokers Age = 40–70 years	Vitamin E (α-tocopherol)	High intake of α-tocopherol (≥14 mg/day) was associated with reduced LC risk in (HR = 0.78, 95% CI = 0.60 to 0.99)
Liu et al. [50]	Europe, North America and Asia	Meta-analysis of six case-control and 16 population-based cohort studies	Total subjects = 15,304 LC current, former and non-smokers	Vitamin D	Vitamin E intake was associated with reduced LC risk in non-smokers only (OR = 0.76, 95% CI = 0.65 to 0.88)

Abbreviation: LC, lung cancer; HR, hazard ratio; RR, relative risk; OR, odds ratio; CI, confidence interval.

## Data Availability

Not applicable.

## Abbreviations

ABCG1	ATP-binding cassette transporter G1
ADC	Adenocarcinoma
AKT	Serine-threonine kinase
AOC	Antioxidant capacity
AP-1	Activator protein-1
BCX	β-cryptoxanthin
BRAF	v-raf murine sarcoma viral oncogene homolog B1
CS	Cigarette smoke
CuZnSOD	Copper-zinc superoxide dismutase
Cx43	Connexin-43
DDR2	Discoidin Domain Receptor Tyrosine Kinase 2
EGFR	Epidermal growth factor receptor
EGFR-TKI	EGFR tyrosine kinase inhibitor
EML4-ALK	Echinoderm microtubule–associate protein like 4/anaplastic lymphoma kinase
ERK	Extracellular signal-regulated kinase
FFQ	Food frequency questionnaire
FGFR	Fibroblast growth factor receptor
GPx	Glutathione peroxidase
GSH	Glutathione
HO-1	Heme oxygenase-1
α7nAChR	homomeric α7 nicotinic acetylcholine receptor
8OHdG	8-hydroxy-20-deoxyguanosine
H2O2	Hydrogen peroxide
IFN-γ	Interferons
IL-	Interleukin
8-iso-PGF_2α_	Isoprostane-8-iso prostaglandin F_2α_
JNK	c-JUN NH2-terminal kinase
KRAS	Kirsten rat sarcoma viral oncogene homolog
LC	Lung cancer
LP	Lipid peroxidation
LSH	Lymphoid-specific helicase
LXRα	Liver X receptor-α
MDA	Malondialdehyde
MMP	Metalloproteinase
MPO	Myeloperoxidase
mTOR	mammalian target of rapamycin
NEIL	Nei like DNA glycosylases
NF-κB	Transcription factor-kappaB
NNAL	Nicotine equivalents and total 4-(methylnitrosamino)-1-(3-pyridyl)-1-butanol
NNK	N-nitrosamines
NO	Nitric oxide
NO2	Nitrogen oxides
NOS	Nitric oxide synthase
NSCLC	Non-small-cell lung cancer
OGG1	8-oxoguanine DNA glycosylase
OS	Oxidative stress
PARP	Poly ADP ribose polymerase
P21	Protein 21
P53	Protein 53
PD-1	Anti-programmed death receptor 1
PI3K	Phosphatidylinositol-3 kinase
PON1	Paraoxonase 1
PPARα	Peroxisome proliferator-activated receptor α
PTEN	Phosphatase and tensin homolog
RA	Retinoid acid
RARβ	RA receptor beta
SIRT1	Sirtuin 1
ROS	Reactive oxygen species
ROS1	ROS Proto-Oncogene 1, Receptor Tyrosine Kinase 1
RCTs	Randomised controlled trials
SCC	Squamous cell carcinoma
SCD-1	Stearoyl-coa desaturase-1
SCLC	Small-cell lung cancer
SHS	Secondhand smoke
SICAM-1	Soluble intercellular adhesion molecule-1
Smad	deca-pentaplegic homolog
SOD	Superoxide dismutase
SOX2	SRY-Box Transcription Factor 2
TNFα	Tumor necrosis factor-α
VEGF	Vascular endothelial growth factor
